# Divergent generative AI pathways in higher education: a parallel mediation analysis of autonomous use and human–machine synergy

**DOI:** 10.3389/fpsyg.2026.1848385

**Published:** 2026-07-09

**Authors:** Yan Cheng, Xinran Liu, Haibo Liu

**Affiliations:** 1 School of General Education, Shandong Huayu University of Technology, Dezhou, China; 2 School of Economics and Management, Shijiazhuang University, Shijiazhuang, China; 3 Dezhou Information Engineering Secondary Vocational School, Dezhou, China; 4 College of Mathematics and Computer, Jilin Normal University, Siping, China

**Keywords:** autonomous learning technology, generative artificial intelligence, human-machine synergy, knowledge application ability, learning motivation, parallel mediation, self-regulated learning

## Abstract

**Introduction:**

The integration of Generative Artificial Intelligence (GenAI) into higher education has raised important questions regarding its influence on student learning outcomes. Drawing on Self-Regulated Learning (SRL) theory, this study examines how learning motivation (LM) relates to knowledge application ability (KAA) through two parallel pathways: human-machine synergy (HMS) and autonomous learning technology (ALT) use.

**Methods:**

Data were collected from 760 Chinese university students and analyzed using structural equation modeling with bias-corrected bootstrapping.

**Results:**

The results indicate that LM has a significant positive direct effect on KAA. Among the two mediating pathways, ALT use demonstrates a small but significant partial mediating effect, whereas the HMS pathway is not statistically significant.

**Discussion:**

These findings suggest that motivated learners may benefit from using GenAI as a self-directed learning support tool, while deeper forms of human-AI collaboration do not necessarily generate stronger knowledge application outcomes. A possible explanation is that excessive cognitive offloading and insufficient algorithmic literacy may weaken the effectiveness of synergistic AI interaction. This study contributes to the literature by highlighting the conditional nature of AI-supported learning and emphasizing the importance of maintaining cognitive autonomy in AI-assisted educational environments.

## 1 Introduction

The deep integration of artificial intelligence into the higher education system is reshaping the structure of learning and teaching practices (Chan and Hu, 2023b; Fawaz et al., 2025; Zhai, 2023). For instance, advanced AI models are increasingly utilized to analyze student engagement in online educational environments (Wang et al., 2025). Building upon this broader technological evolution, the specific emergence of Generative Artificial Intelligence (GenAI) has triggered a shift in how university students acquire information, process concepts, and apply complex knowledge. Unlike traditional static digital learning resources, contemporary AI technologies possess real-time computation and natural language processing capabilities that allow them to act as dynamic conversational agents (Fel et al., 2025; Kasneci et al., 2023). Consequently, the academic community has engaged in ongoing debates regarding the actual impact of AI on cognitive development. While some scholars argue that AI tools provide necessary scaffolding to enhance individual learning performance (Cheng et al., 2025), others warn about the risks of cognitive offloading where students delegate critical thinking processes to algorithms (Hu et al., 2022).

Within this ongoing debate, a limitation in current literature is the tendency to treat AI usage as a homogeneous behavior. Most empirical studies measure the frequency or general acceptance of AI tools without distinguishing the qualitative differences in how students interact with these systems (Bernabei et al., 2023). While student-AI interactions theoretically exist on a continuous spectrum of cognitive engagement, learners in high-stakes academic environments tend to adopt them as distinct functional strategies based on immediate task demands. At one end of this spectrum, students utilize AI as an autonomous learning technology (ALT) for discrete, efficiency-driven tasks, such as checking grammatical structures, extracting summaries from extensive literature, or generating basic code syntax (Derouech et al., 2025). At the other end, students engage in human-machine synergy (HMS), treating the AI as a collaborative partner. This synergistic mode involves iterative prompt engineering, debating complex theoretical concepts, and co-constructing logical frameworks (Nasr et al., 2025). Although these two interaction models likely produce vastly different cognitive outcomes, empirical evidence comparing their parallel mechanisms remains scarce. Therefore, a clearer distinction between efficiency-oriented AI use and deeper human-AI collaboration is necessary for understanding the educational consequences of GenAI-supported learning.

To address this critical research gap, it is essential to understand what drives students to choose a specific mode of interaction and how this choice ultimately affects their academic capabilities (Wang, 2024). According to Self-Regulated Learning theory, a learner's internal psychological state is the primary driver of their behavioral strategies and subsequent performance outcomes (Chan, 2023a). Learning motivation serves as the fundamental psychological driver that initiates and sustains educational engagement (Galindo-Domínguez et al., 2026; Su and Yang, 2023). Highly motivated students are theoretically more likely to actively seek out resources and adopt advanced learning strategies. However, in an AI-ubiquitous environment, it remains unclear whether learning motivation drives students toward deep synergistic collaboration, efficiency-driven autonomous technology use, or both. Furthermore, the downstream effects of these two pathways on knowledge application ability require rigorous statistical examination (Sun et al., 2025).

Therefore, this study aims to construct and validate a parallel mediation model to explore the two pathways linking learning motivation and knowledge application ability. Specifically, we investigate human-machine synergy and autonomous learning technology use as two parallel mediators (Juárez et al., 2026). By examining these two parallel pathways, this research seeks to answer a fundamental question: How does learning motivation enhance knowledge application, and what roles do synergistic and autonomous AI interactions play in this process? This question shifts the focus from whether students use AI to how they regulate different forms of AI use during learning.

This study makes three contributions to the literature. First, by distinguishing between human-machine synergy (HMS) and autonomous learning technology (ALT) use, this study highlights the heterogeneous and potentially divergent nature of AI-supported learning behaviors in higher education, moving beyond generalized assumptions of technology acceptance (Adiyono et al., 2025; Karimova et al., 2025). Second, the study extends Self-Regulated Learning (SRL) theory into the algorithmic era by demonstrating that different forms of AI interaction may activate distinct cognitive regulatory mechanisms rather than producing uniformly beneficial learning outcomes (Azevedo and Gašević, 2019). Third, this research introduces an important boundary-condition perspective to AI-assisted learning by suggesting that deeper human-AI collaboration does not necessarily translate into stronger knowledge application. Instead, the educational value of AI interaction may depend on learners' ability to maintain cognitive autonomy and critically regulate algorithmic assistance during the learning process (Islam et al., 2025). These findings provide practical implications for educators seeking to design pedagogical interventions that encourage productive AI use while minimizing excessive cognitive offloading and overreliance on algorithmic outputs.

## 2 Literature review and hypotheses development

### 2.1 Theoretical foundation: self-regulated learning theory in the algorithmic era

Self-Regulated Learning theory is a foundational framework for understanding how learners achieve academic goals. It posits that learners actively govern their internal psychological states and external behavioral strategies. In its traditional conceptualization, the SRL process is modeled as a cyclical mechanism comprising three distinct phases including forethought, performance control, and self-reflection (Chan, 2023a). During the forethought phase, learners analyze task demands and activate their internal motivational beliefs. This psychological preparation subsequently dictates the deployment of specific cognitive strategies during the performance control phase (Cassidy, 2011). Finally, learners evaluate their academic outcomes against their initial goals during the self-reflection phase, which in turn influences future motivational states (Wang et al., 2017). While this theory has been extensively validated in traditional and early digital learning environments, the rapid integration of Generative Artificial Intelligence disrupts the established boundaries of performance control (Kasneci et al., 2023).

In conventional educational settings, learners rely predominantly on their internal cognitive resources or peer interactions to overcome academic friction. However, in an AI-ubiquitous environment, the locus of cognitive processing is no longer confined to the human mind (Wei et al., 2025). Contemporary AI tools possess advanced natural language processing and real-time computational capabilities that allow them to act as dynamic intellectual agents rather than static repositories of information (Su and Yang, 2023). This technological paradigm shift requires an evolution in SRL theory from purely internal regulation to hybrid human-machine synergy (Borzanović et al., 2026; Juárez et al., 2026). Hybrid regulation posits that modern learners must synchronously monitor their own cognitive understanding while critically evaluating the algorithmic output generated by the machine (Chan, 2023a).

Viewed through this updated theoretical lens, the interaction between university students and AI cannot be treated as a homogeneous behavioral construct. Instead, it bifurcates into distinct regulatory strategies. Within the performance control phase of the SRL framework, the integration of AI creates a hybrid regulatory environment. Here, autonomous learning technology use (ALT) functions as an efficiency-driven, lower-cognitive-load regulatory strategy, focusing on task management, task delegation, and resource allocation (Heung and Chiu, 2025). In this study, ALT specifically refers to learner-directed and instrumental AI use for bounded academic tasks, where students retain control over learning goals and knowledge integration. Conversely, human-machine synergy (HMS) represents a deep, iterative regulatory strategy that requires continuous metacognitive monitoring, iterative prompting, and reciprocal knowledge construction (Tekir, 2026). HMS therefore refers to a co-constructive mode of interaction in which students treat AI as a dialogic partner for idea refinement and complex problem solving. By explicitly situating these two behavioral constructs within the performance control phase, the present study establishes a theoretical pathway to explore how initial motivational states distinctly activate these interaction patterns, ultimately leading to divergent higher-order cognitive outcomes.

### 2.2 Learning motivation and human-machine synergy patterns

Learning motivation constitutes the primary psychological driver within the forethought phase of self-regulation. It represents the internal drive that compels students to initiate academic activities, sustain cognitive effort, and pursue mastery over complex subject matter (Wang et al., 2023). Extensive literature in educational psychology consistently demonstrates that motivation is a critical antecedent for technology adoption (Cheng et al., 2025). However, the specific behavioral manifestation of this technological adoption is contingent upon both the affordances of the tool and the learner's underlying psychological intent (Chiu, 2024).

From a theoretical perspective, a positive association may emerge between learning motivation and human-machine synergy. Developing a collaborative dynamic with an algorithmic agent may require considerable cognitive investment and sustained engagement. Rather than passively accepting the initial output provided by the system, learners aiming for synergy are expected to engage in sophisticated prompt engineering, identify logical inconsistencies, and iteratively refine conversational parameters to explore complex concepts deeply (Su and Yang, 2023). This iterative evaluation process demands a high degree of academic persistence. Therefore, it is theorized that students exhibiting strong learning motivation are more likely to possess the requisite psychological energy to sustain this demanding interaction (Wang et al., 2023). They tend to perceive the AI not merely as an automated answering machine, but as a potential intellectual partner (Kasneci et al., 2023). Consequently, elevated levels of internal motivation may provide the driving force necessary to overcome the cognitive friction inherent in human-machine synergy (Borzanović et al., 2026). Nevertheless, the relationship between learning motivation and deep human–AI collaboration remains theoretically uncertain in educational contexts. This uncertainty indicates that motivation may provide the psychological basis for engaging with AI, but it may not be sufficient to ensure high-quality human-AI collaboration without critical regulation.

Hypothesis 1 (H1): Learning motivation has a significant positive effect on human-machine synergy.

However, the educational benefits of human-machine synergy may not be universally guaranteed. Emerging research suggests that intensive collaboration with generative AI can also introduce risks of cognitive offloading, reduced independent reasoning, and overreliance on algorithmic outputs (Ivanov, 2023; Galindo-Domínguez et al., 2026). In such cases, learners may perceive themselves as engaging in deep collaborative learning while actually delegating substantial cognitive processing to the AI system. Consequently, the effectiveness of HMS may depend on whether students maintain sufficient cognitive autonomy and algorithmic literacy during interaction with AI tools.

In parallel, learning motivation is theorized to positively influence the frequency and intensity of autonomous learning technology use. While ALT involves a comparatively shallower form of cognitive engagement, it remains a highly rational and proactive academic management strategy (Heung and Chiu, 2025). Highly motivated learners are characteristically resourceful. They actively seek mechanisms to optimize their learning efficiency, bypass redundant administrative tasks, and resolve minor procedural hurdles rapidly (Su and Yang, 2023). In contemporary university settings, utilizing AI to rapidly summarize voluminous literature, correct structural grammatical errors, or generate fundamental code syntax represents a pragmatic approach driven by the motivation to maximize academic output (Chan and Hu, 2023b). The instant gratification and operational accessibility provided by modern algorithmic tools make them attractive to students who are motivated to maintain high performance metrics while managing strict temporal constraints (Heung and Chiu, 2025). Therefore, increased motivation naturally translates into a higher propensity to leverage AI as an autonomous instrumental tool.

Hypothesis 2 (H2): Learning motivation has a significant positive effect on autonomous learning technology use.

### 2.3 The divergent pathways to knowledge application ability

Knowledge application ability represents a higher-order cognitive skill that extends beyond the mere retention of factual declarative knowledge. It entails the capacity to synthesize diverse concepts, adapt theoretical frameworks to novel empirical contexts, and resolve unstructured real-world problems (Yang et al., 2024). In the context of algorithmic learning environments, the structural impact of AI interaction on this cognitive capacity is theorized to depend heavily on the nature of the interaction mechanism (Tekir, 2026).

Theoretically, human-machine synergy may contribute to the development of knowledge application ability by encouraging learners to participate more actively in the knowledge construction process rather than remaining passive recipients. Recent investigations suggest that constructive interaction during human-AI collaboration can facilitate creative problem-solving (Wei et al., 2025). Through synergistic interaction with the AI, learners are often prompted to externalize their tacit knowledge through precise query formulation (Zhai, 2023). Furthermore, this interactive mode ideally requires them to evaluate algorithmic suggestions against their own domain expertise, identifying potential biases or logical fallacies before internalizing the information (Chan, 2023a). This continuous cycle of critical evaluation is believed to activate the core metacognitive faculties necessary for complex knowledge application. By supporting independent critical thinking and conscious control over the learning process, HMS may support higher-order learning under certain conditions (Yang et al., 2024). However, recent studies also suggest that the educational effectiveness of deep human-AI collaboration may depend on learners' ability to maintain independent cognitive engagement during interaction with algorithmic systems. Without sufficient cognitive regulation, synergistic AI use may inadvertently encourage excessive reliance on algorithmic reasoning rather than active knowledge construction (Ivanov, 2023; Peng et al., 2026). Thus, the key issue is not whether students interact deeply with AI, but whether they remain cognitively active and critically engaged during such interaction. Overall, prior evidence regarding the educational effectiveness of deep human–AI collaboration remains mixed, suggesting the need for further empirical examination.

Hypothesis 3 (H3): Human-machine synergy has a significant positive effect on knowledge application ability.

Conversely, the relationship between autonomous learning technology use and knowledge application ability is theorized to operate through a distinct cognitive mechanism grounded in Cognitive Load Theory. The acquisition of complex knowledge often imposes a substantial intrinsic cognitive load on novice learners. In this context, utilizing ALT functions primarily as a load-reduction strategy. By delegating lower-order processing tasks to the algorithmic system, students can allocate their limited working memory capacity more efficiently (Kasneci et al., 2023). For example, delegating the translation of foreign literature or the summarization of basic historical facts allows learners to redirect their cognitive resources toward analytical synthesis and critical application (Chan and Hu, 2023b). Although this mode of interaction carries the risk of excessive cognitive offloading, the strategic use of AI to establish an informational baseline is anticipated to facilitate the foundational learning phase (Ivanov, 2023; Su and Yang, 2023). This expanded accessible knowledge base subsequently provides the necessary information schemas from which students can draw when executing higher-order application tasks.

Hypothesis 4 (H4): Autonomous learning technology use has a significant positive effect on knowledge application ability.

### 2.4 Parallel mediation and the competitive hypothesis

Grounded in Self-Regulated Learning (SRL) theory, this study posits that human-machine synergy (HMS) and autonomous learning technology (ALT) use function as parallel mediating pathways. Motivation, as a latent psychological construct, typically requires translation into concrete regulatory behaviors to meaningfully influence cognitive outcomes (Humble, 2024).

The first mechanism operates through a synergistic pathway. Driven by intrinsic motivation for academic mastery, students are more likely to initiate iterative dialogues with AI systems. This active interaction demands critical analysis, evaluation, and synthesis, which, in turn, are anticipated to facilitate the capacity to apply knowledge across novel contexts (Kothari and Fernando, 2024).

H5a: Human-machine synergy significantly mediates the relationship between learning motivation and knowledge application ability.

The second parallel mechanism operates through an efficiency-driven pathway. Motivation may also drive students to optimize their learning processes by strategically deploying AI as an autonomous tool. By mitigating cognitive overload, this delegation preserves cognitive resources, thereby providing the foundational informational schemas required for subsequent knowledge application tasks (Su and Yang, 2023).

H5b: Autonomous learning technology use significantly mediates the relationship between learning motivation and knowledge application ability.

**Figure 1:**
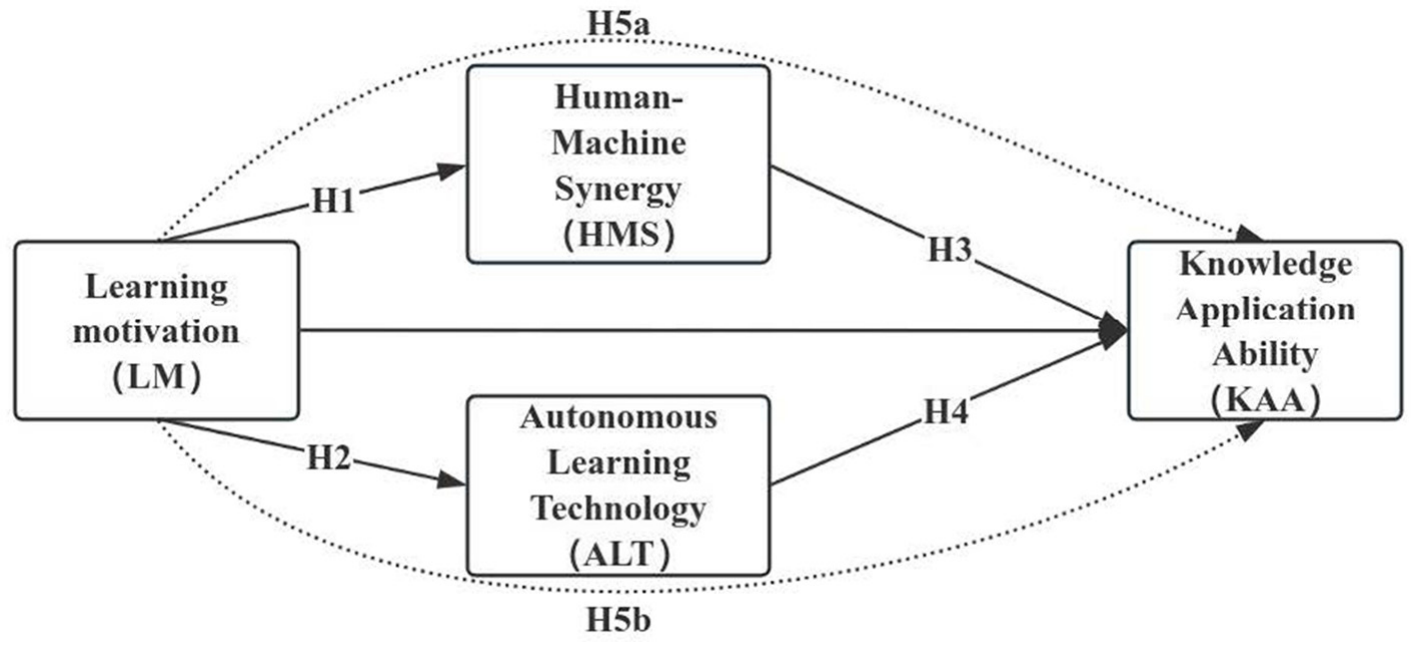
The conceptual model and hypothesized paths of the current study. *Solid lines represent hypothesized direct effects, while indirect paths indicate mediation relationships*.

Based on the theoretical deduction and research hypotheses presented above, the conceptual model of this study is illustrated in Figure 1.

## 3 Methodology

### 3.1 Sample and data collection

This study employed a quantitative research design utilizing a cross-sectional survey method. Data were collected from university students enrolled in higher education institutions. These institutions were selected due to their active promotion of digital learning infrastructure and generative artificial intelligence tools. Prior to data collection, the research protocol received approval from the relevant institutional review board. All participants provided explicit informed consent. They were thoroughly informed about the voluntary nature of the study and the confidentiality of their responses.

A purposive sampling technique was utilized to recruit students. Specifically, a strict inclusion criterion was applied: participants were required to have at least 3 months of active experience using generative artificial intelligence tools for academic purposes to ensure the validity and relevance of their responses. The survey was distributed via a professional online questionnaire platform. We implemented quality control measures to ensure data integrity. Researchers strategically embedded attention check items within the survey. Responses failing these attention checks were immediately discarded. Questionnaires displaying identical answers across all items or completion times under 2 min were also excluded from the final dataset.

Following these data cleaning procedures, the final valid sample comprised 760 participants, with a balanced gender distribution (46.4% male, *n* = 353; 53.6% female, *n* = 407). This balanced gender ratio effectively minimizes potential gender-based biases in technology adoption behaviours. Furthermore, this sample size adequately satisfies the statistical requirements for structural equation modelling. Methodological guidelines generally recommend a minimum of 200 cases or a ratio of 10 cases per observed variable for robust parameter estimation. Our final valid sample of 760 participants exceeds the recommended threshold. This robust sample size provides adequate statistical power for the subsequent parallel mediation analyses.

### 3.2 Instruments

In this study, the survey instrument consisted of two main sections. The first section collected participants' demographic information consisting solely of gender. The second section comprised measurement items for the four core latent variables in the research model. These variables are learning motivation, human-machine synergy, autonomous learning technology use, and knowledge application ability.

All measurement items were adapted from well-validated literature to ensure content validity. Minor wording modifications were made to tailor the items to the specific context of university students learning with generative artificial intelligence technologies. Because the questionnaire was originally developed in English and administered to Chinese students, the translation followed Brislin (1970) classic back-translation model. Specifically, two bilingual researchers independently translated the items from English to Chinese, and a third expert back-translated them to English to ensure semantic equivalence and mitigate cross-cultural bias. To further ensure content validity, three experts in educational psychology and applied linguistics were invited to review the questionnaire. They evaluated the clarity, relevance, and appropriateness of each item. Based on their feedback, minor revisions were made to improve wording and clarity. Before the formal survey, a pilot test was conducted with a small sample of university students to assess the comprehensibility and feasibility of the instrument. The results indicated that the questionnaire was clear and easy to understand. Several minor adjustments were made accordingly. The full measurement items for each construct are presented in Table 1. After data collection, the reliability and validity of the measurement scales were thoroughly assessed. All constructs were measured using a five-point Likert scale ranging from 1 (strongly disagree) to 5 (strongly agree).

**Table 1:** Full measurement items for constructs.

Construct (and Abbreviation)	Source(s)	Measurement Items (with item codes)
**Learning Motivation (LM)**	Adapted from Lo et al. (2022)	**LM1:** I am highly motivated to explore new knowledge in my academic studies.
		**LM2:** I feel a strong internal drive to master the content in my courses.
		**LM3:** I find learning new things in my field of study genuinely interesting and enjoyable.
**Human-Machine Synergy (HMS)**	Adapted from Li et al. (2023b)	**HMS1:** I closely collaborate with artificial intelligence tools to jointly optimize my learning processes.
		**HMS2:** I use AI as a thinking partner, engaging in back-and-forth dialogue to refine complex ideas.
		**HMS3:** I feel that my own ideas and the AI's suggestions combine to create outcomes that are better than what I could produce alone.
**Autonomous Learning Technology (ALT) Use**	Adapted from Teo et al. (2010)	**ALT1:** I independently use artificial intelligence technologies to plan and monitor my learning progress.
		**ALT2:** I use AI tools to efficiently handle routine tasks (e.g., summarizing articles, checking grammar) so I can focus on more complex thinking.
		**ALT3:** When I encounter a learning problem, I proactively use AI to find information and solve it on my own.
**Knowledge Application Ability (KAA)**	Adapted from Lo et al. (2022)	**KAA1:** I can effectively apply the knowledge acquired from my studies to solve real-world problems.
		**KAA2:** I am good at adapting theoretical knowledge to fit new or unfamiliar situations.
		**KAA3:** I can synthesize information from various sources to develop a coherent solution to a problem.
		**KAA4:** When facing a complex task, I can draw upon my academic knowledge to guide my actions.

#### 3.2.1 Learning motivation

Learning motivation was adapted from the validated scale developed by Lo et al. (2022). This construct was measured using items assessing students' intrinsic drive and active engagement in their academic pursuits. A sample item is: “I am highly motivated to explore new knowledge in my academic studies.” The scale demonstrated acceptable reliability.

#### 3.2.2 Human-machine synergy

Human-machine synergy was adapted from the scale developed by Li et al. (2023b). Originally designed for employee work contexts, the items were contextualized to fit the academic learning environment. This construct evaluated the degree of deep collaboration and mutual cognitive enhancement between students and generative artificial intelligence tools. A sample item is: “I closely collaborate with artificial intelligence tools to jointly optimize my learning processes.” The scale showed good reliability.

#### 3.2.3 Autonomous learning technology use

Autonomous learning technology use was adapted from the self-directed learning with technology scale developed by Teo et al. (2010). This construct included items focusing on students' independent management and self-directed utilization of artificial intelligence technologies for academic purposes. A sample item is: “I independently use artificial intelligence technologies to plan and monitor my learning progress.” The scale demonstrated strong reliability.

#### 3.2.4 Knowledge application ability

Knowledge application ability was derived from the scale validated by Lo et al. (2022). This variable was measured using items evaluating students' capacity to effectively transfer theoretical knowledge into practical problem-solving scenarios. A sample item is: “I can effectively apply the knowledge acquired from my studies to solve real-world problems.” The scale demonstrated strong reliability.

### 3.3 Data analysis

The empirical data collected in this study were systematically analyzed utilizing the statistical software packages SPSS 27.0 and AMOS 28.0. A rigorous sequential analytical strategy was adopted to examine the measurement and structural models. First, preliminary analyses were executed to compute descriptive statistics and assess the distributional characteristics of the dataset. Given the self-reported nature of the questionnaire, Common Method Bias was evaluated through a tri-methodological approach. This comprehensive assessment included the Harman single-factor test, a Confirmatory Factor Analysis competitive model comparison, and the Unmeasured Latent Method Construct technique to ensure that systematic variance did not artificially inflate the observed relationships. Second, the measurement model was validated to confirm its psychometric robustness. Internal consistency and reliability were established by calculating Cronbach α coefficients and Composite Reliability values. Convergent validity was confirmed by examining the standardized item loadings and the Average Variance Extracted for each latent construct. Discriminant validity was subsequently verified by ensuring that the square root of the Average Variance Extracted for every construct strictly exceeded its corresponding inter-construct correlations. Third, structural equation modeling was employed to examine the proposed directional relationships and test the direct hypotheses. The overall adequacy of the structural model was determined using standard goodness-of-fit indices. Finally, to investigate the specific parallel mediating roles of human-machine synergy and Autonomous Learning Technology, a bias-corrected non-parametric bootstrapping procedure encompassing 5,000 resamples was utilized. The statistical significance of the indirect effects was confirmed if the 95 percent confidence intervals strictly excluded zero.

## 4 Results

### 4.1 Common method bias assessment

Data for this study were collected through a cross-sectional self-report survey. Common method bias was therefore examined to ensure data validity. Three distinct statistical approaches were employed to address this concern (Su et al., 2025).

First, Harman's single-factor test was conducted using exploratory factor analysis. An unrotated principal component analysis was performed on all measurement items. The results showed that the first major factor accounted for 38.067% of the total variance. This value falls well below the widely accepted critical threshold of 50%.

Second, a single-factor confirmatory factor analysis competitive model comparison was executed. The hypothesized four-factor measurement model was compared against a single-factor model (Cheng and Liu, 2026). In the single-factor model, all items were forced to load onto one overarching factor. Table 2 presents the results of this comparison. The four-factor model demonstrated a significantly better fit than the single-factor model. The chi-square difference between the two models was significant (Δχ^2^ = 3498.76, Δdf = 6, *p* < 0.001). This confirms that the constructs are distinct and not driven by a single underlying method factor.

**Table 2:** Changes of CFA comparison model.

Model	χ^2^	df	Δχ^2^	Δdf	*p*
One-factor	3,620.021	65	3,498.76	6	< 0.001
Four-factor	121.261	59			

Third, the unmeasured latent method construct test was applied for further verification. A common method factor was introduced into the baseline four-factor model (M1) to create an updated method factor model (M2). The fit indices of these two models were then systematically compared. Table 3 illustrates the changes in the goodness-of-fit indices. The observed variations were minimal. Specifically, the changes included ΔGFI = 0.011, ΔCFI = 0.006, ΔTLI = 0.006, ΔRMSEA = −0.012, and ΔSRMR = −0.008. All these fluctuations fall strictly below the rigorous threshold of 0.05. The inclusion of the latent method factor did not substantially improve the overall model fit (Liu and Cheng, 2026).

**Table 3:** ULMC test results.

Model	χ^2^	df	χ^2^/df	GFI	CFI	TLI	RMSEA	SRMR	ΔGFI	ΔCFI	ΔTLI	ΔRMSEA	ΔSRMR
M1	121.261	59	2.055	0.975	0.991	0.989	0.037	0.0258	0.011	0.006	0.006	−0.012	−0.008
M2	68.64	47	1.46	0.986	0.997	0.995	0.025	0.0177					

Collectively, the results from these three diagnostic tests suggest that common method bias does not appear to be a major concern in this dataset, thus supporting the robustness of the data for subsequent analysis.

### 4.2 Measurement model assessment

Confirmatory factor analysis was conducted to evaluate the reliability and validity of the measurement model. The evaluation focused on internal consistency convergent validity and discriminant validity. Table 4 presents the detailed results of the measurement model assessment.

**Table 4:** Reliability and convergent validity of the measurement model.

Constructs	Indicators	*B*	*SE*	*z*	*p*	β	α	CR	AVE
KAA	KAA1	1				0.883	0.946	0.947	0.816
	KAA2	1.029	0.027	38.306	^***^	0.917			
	KAA3	1.032	0.026	39.391	^***^	0.928			
	KAA4	0.994	0.028	35.385	^***^	0.885			
HMS	HMS1	1				0.610	0.801	0.808	0.589
	HMS2	1.209	0.073	16.553	^***^	0.808			
	HMS3	1.277	0.076	16.866	^***^	0.861			
LM	LM1	1				0.821	0.899	0.902	0.755
	LM2	1.046	0.035	29.814	^***^	0.919			
	LM3	0.997	0.035	28.125	^***^	0.864			
ALT	ALT1	1				0.867	0.897	0.897	0.744
	ALT2	1.02	0.033	30.562	^***^	0.881			
	ALT3	0.96	0.033	28.678	^***^	0.840			

^***^p<*0.001. LM, Learning Motivation; HMS, Human-Machine Synergy; ALT, Autonomous Learning Technology; KAA, Knowledge Application Ability*.

Reliability was assessed using Cronbach's α and composite reliability metrics. The widely accepted threshold for both indicators is 0.70. As shown in the analytical results, the Cronbach's α values for all four constructs ranged from 0.801 to 0.946. Furthermore, the composite reliability values ranged from 0.808 to 0.947. These values substantially exceed the recommended threshold. This confirms that the measurement model possesses excellent internal consistency.

Convergent validity was evaluated through item loadings and the average variance extracted metric. Statistical standards require standardized item loadings to be significant and ideally above 0.60. The average variance extracted should exceed 0.50 (Peng et al., 2026). The data analysis showed that all item loadings were significant (*p* < 0.001). The standardized loadings ranged from 0.610 to 0.928. Concurrently, the average variance extracted values for all constructs ranged from 0.589 to 0.816. These results demonstrate strong convergent validity across all theoretical constructs.

Discriminant validity was verified using the strictly defined Fornell-Larcker criterion. This approach requires the square root of the average variance extracted for each construct to be strictly greater than its correlation coefficients with any other construct. Table 5 illustrates the discriminant validity results. The diagonal elements represent the square root of the average variance extracted. The lowest diagonal value is 0.767 for HMS. This value remains higher than the maximum off-diagonal correlation coefficient which is 0.685 between HMS and ALT. The measurement model thereby satisfies the Fornell-Larcker criterion. Discriminant validity is established.

**Table 5:** Discriminant validity.

Dimension	ALT	LM	HMS	KAA
ALT	**0.863**			
LM	0.255	**0.869**		
HMS	0.685	0.011	**0.767**	
KAA	0.194	0.574	−0.006	**0.903**

*The diagonal bold part is the square root of the AVE. LM, Learning Motivation; HMS, Human-Machine Synergy; ALT, Autonomous Learning Technology; KAA, Knowledge Application Ability*.

### 4.3 Path analysis

Structural equation modeling was employed to empirically examine the proposed conceptual model. The maximum likelihood estimation method was utilized to calculate the path coefficients and assess the significance of the hypothesized relationships. Table 6 presents the comprehensive results of the path analysis.

**Table 6:** Path coefficients and hypothesis testing results of the structural model.

Hypothesized relationship	β	*B*	*SE*	*z*	*p*
H1: LM → HMS	−0.011	−0.01	0.035	−0.293	0.769
H2: LM → ALT	0.229	0.221	0.034	6.48	^***^
H3: HMS → KAA	−0.042	−0.049	0.035	−1.375	0.169
H4: ALT → KAA	0.083	0.097	0.037	2.647	0.008
LM → KAA	0.520	0.591	0.035	16.67	^***^

^***^p<*0.001. LM, Learning Motivation; HMS, Human-Machine Synergy; ALT, Autonomous Learning Technology; KAA, Knowledge Application Ability*.

The analysis evaluated the direct structural paths among the latent variables. The results indicate that the relationship between learning motivation and human-machine synergy is not statistically significant (β = −0.011, *p* = 0.769). Therefore, H1 is rejected. Conversely, learning motivation exhibits a significant positive effect on autonomous learning technology (β = 0.229, *p* < 0.001). This finding provides empirical support for H2.

Furthermore, the hypothesized path from human-machine synergy to knowledge application ability lacks statistical significance (β = −0.042, *p* = 0.169). Consequently, H3 is not supported by the current data. In contrast, autonomous learning technology demonstrates a significant positive impact on knowledge application ability (β = 0.083, *p* = 0.008). This supports H4.

Additionally, the analysis reveals that the direct path from learning motivation to knowledge application ability is significant and positive (β = 0.520, *p* < 0.001). This indicates a strong baseline relationship between these two core constructs. The structural model helps explain the different roles of AI usage patterns in higher education learning.

### 4.4 Mediation effect testing

To test the parallel mediating roles of human-machine synergy (HMS) and autonomous learning technology (ALT) between learning motivation (LM) and knowledge application ability (KAA), a bias-corrected percentile bootstrapping method with 5,000 resamples was employed to calculate the 95% confidence intervals (CIs). The results of the mediation analysis are presented in Table 7.

**Table 7:** Parallel mediating effects of HMS and ALT.

Path relationship	Point estimate	SE	95% CI (Lower)	95% CI (Upper)
LM → HMS → KAA(IE1)	0.001	0.003	−0.004	0.010
LM → ALT → KAA(IE2)	0.021	0.013	0.001	0.051
LM → KAA(DE)	0.591	0.040	0.511	0.668
Total=(IE1+IE2+DE)	0.613	0.037	0.537	0.683
IE1/Total	0.001	0.005	−0.006	0.016
IE2/Total	0.035	0.022	0.001	0.084

*LM, Learning Motivation; HMS, Human-Machine Synergy; ALT, Autonomous Learning Technology; KAA, Knowledge Application Ability. IE, Indirect Effect; DE, Direct Effect*.

First, regarding the mediating pathway of human-machine synergy (IE1: LM → HMS → KAA), the point estimate for the indirect effect was 0.001. The 95% CI ranged from−0.004 to 0.010. Because this confidence interval includes zero, the mediating effect of HMS is not statistically significant. Therefore, H5a is not supported.

Second, regarding the mediating pathway of autonomous learning technology (IE2: LM → ALT → KAA), the point estimate for the indirect effect was 0.021. The 95% CI ranged from 0.001 to 0.051. Because this interval does not encompass zero, the mediating effect of ALT is statistically significant. Furthermore, the direct effect (DE) of LM on KAA remained significant (estimate = 0.591, 95% CI = [0.511, 0.668]). This indicates that ALT plays a partial mediating role between learning motivation and knowledge application ability, accounting for 3.5% (IE2/Total = 0.035) of the total effect. Thus, H5b is supported.

## 5 Discussion

### 5.1 Overview of the findings

As the rapid integration of generative artificial intelligence alters higher education, a reevaluation of how students regulate their learning is required. Based on self-regulated learning theory, this study proposed a parallel mediation model to investigate how learning motivation transforms into knowledge application ability through two distinct technological pathways: human-machine synergy and autonomous learning technology use. Our empirical results showed a direct positive effect of learning motivation on knowledge application ability, reinforcing the premise that intrinsic motivation is a fundamental antecedent for cognitive development (Zheng et al., 2024; Lo et al., 2022). However, the main contribution of this study comes from the mediation analysis. The data demonstrated an asymmetrical mediating pattern: ALT use significantly mediated the relationship between learning motivation and knowledge application ability, supporting H5b. Conversely, the mediating pathway through HMS was statistically non-significant, leading to the rejection of H5a. This finding suggests that the theoretical expectations of the HMS pathway may be constrained by practical challenges. In this context, although the mediating effect size of ALT was relatively modest, the finding still suggests that strategically regulated AI use may contribute to knowledge application under AI-supported learning conditions. These findings challenge generalized assumptions regarding the uniform effectiveness of different AI interaction models and provide empirical insights into the actual dynamics of AI-assisted learning.

### 5.2 The mediating roles of HMS and ALT

The significant mediating role of autonomous learning technology (ALT) suggests that motivated students may benefit from using generative AI as a self-directed academic support tool rather than as a substitute for cognitive processing. In this mode of interaction, learners remain primarily responsible for setting goals, evaluating information quality, and integrating AI-generated outputs into their own understanding. Generative AI therefore functions more as a supplementary cognitive scaffold than as an independent problem-solving agent (Bai et al., 2026; Wang and Long, 2025). This finding aligns with the core assumptions of self-regulated learning theory, which emphasizes learners' active control over learning strategies and cognitive monitoring (Yang, 2026; Jazim et al., 2025). Although the indirect effect size of ALT was relatively small, the result nonetheless provides empirical evidence that strategically regulated AI use may contribute to baseline knowledge application in higher education contexts.

In contrast, the non-significant mediating role of human-machine synergy (HMS) suggests that deeper forms of AI collaboration do not automatically produce stronger learning outcomes. While prior literature often assumes that collaborative interaction with AI enhances higher-order cognition, the present findings indicate that such benefits may be conditional rather than universal. One plausible explanation involves cognitive offloading and competence overestimation (Pratiwi et al., 2025; Qi et al., 2026). During intensive interaction with generative AI systems, students may be more likely to transfer analytical and reasoning tasks to the algorithm itself while retaining only superficial supervisory roles. Under these circumstances, learners may develop a perceived sense of understanding because the AI produces coherent and sophisticated outputs with relatively limited human cognitive effort.

Rather than facilitating deep conceptual restructuring, excessive reliance on algorithmic collaboration may weaken the active mental processing required for genuine knowledge internalization. Recent studies similarly caution that generative AI can sometimes reduce independent critical engagement and foster cognitive dependency when pedagogical guidance is insufficient (Galindo-Domínguez et al., 2026; Wu et al., 2026). Therefore, the present findings suggest that HMS may only become educationally effective when learners possess adequate algorithmic literacy and maintain conscious cognitive regulation during interaction with AI systems. Without these conditions, deeper collaboration with AI may improve task completion efficiency without necessarily strengthening knowledge application ability. It should also be noted that cognitive offloading, algorithmic literacy, and metacognitive regulation were not directly measured in the present study; therefore, this explanation should be regarded as a theoretically grounded account that requires further empirical testing.

This behavioral reality offers a plausible explanation for why the synergistic approach, unlike the autonomous use of AI, did not yield a statistically significant mediating effect in our model. This conclusion aligns with recent systematic reviews. These reviews emphasize that the educational benefits of AI on student engagement are heavily contingent upon the intervention of appropriate teaching methods (Long et al., 2026).

### 5.3 Theoretical implications

This study makes three substantial contributions to the literature on educational technology and psychology. First, it extends self-regulated learning theory into the algorithmic era by structurally distinguishing between autonomous tool use and synergistic collaboration. Unlike previous models that often treated technology usage as a single construct, conceptualizing synergy and autonomous use as distinct parallel pathways provides a granular understanding of AI interaction, showing how different interaction modes can yield divergent cognitive outcomes (Huang et al., 2024; Sung and Huang, 2026).

Second, this research introduces essential boundary conditions for human-AI collaboration in education. While human-machine synergy is effective in industrial contexts for maximizing productivity, our findings suggest that increased task productivity through AI collaboration does not necessarily translate into deeper learning or stronger knowledge application. By showing that HMS did not significantly predict knowledge application ability in the present model, this study suggests that deep human-AI interaction may not automatically facilitate knowledge internalization, especially when cognitive regulation is insufficient (Qi et al., 2026; Xie et al., 2025).

Third, this study shifts the academic focus from technology adoption to interaction quality. Because the synergistic model reveals a disconnected pathway between motivation and knowledge application, our results suggest that intrinsic motivation alone may be insufficient to guarantee positive learning outcomes in AI-supported environments. Specifically, positive learning outcomes are compromised if pedagogical designs allow students to outsource their critical thinking to large language models (Balaskas et al., 2025). This study challenges the widely held assumption that deeper human-AI interaction necessarily leads to better learning outcomes.

### 5.4 Practical implications

The findings offer actionable guidelines for university administrators, curriculum designers, and educators. First, educators may need to reconsider traditional assessment strategies to mitigate the risk of cognitive offloading. As evidenced by the success of the autonomous pathway, meaningful knowledge application is more likely to occur when students maintain cognitive agency. Therefore, instructional designs should explicitly require students to demonstrate their thinking process, compelling educators to evaluate how students processed, critiqued, and modified AI-generated outputs. Process-oriented assessments and oral defenses should replace traditional take-home essays, helping prevent excessive dependence on algorithmic outputs (Pratiwi et al., 2025).

Second, universities must prioritize the development of critical AI literacy. Guided by the successful mediation of autonomous learning technology, institutions should teach students how to use generative AI as a self-directed tool. While students can use AI for brainstorming or generating formative feedback, the synthesis and analytical phases must remain strictly human efforts. Institutional policies should provide clear ethical guidelines. Simultaneously, educators must train students in advanced prompt engineering, ensuring that students dictate the algorithmic output rather than passively accepting it (Bai et al., 2026; Li, 2023a).

Third, curriculum designers should embed AI-supported learning activities into formal course structures rather than treating AI use as an informal or uncontrolled learning practice. For example, courses may include AI-use reflection logs, prompt revision assignments, AI-output critique tasks, and classroom discussions on the limitations of algorithmic reasoning. These activities can make students' AI-assisted learning processes visible and help instructors evaluate not only final learning products but also how students use, question, revise, or reject AI-generated outputs. In this way, curriculum design can shift AI use from passive dependence to regulated, reflective, and pedagogically meaningful engagement.

### 5.5 Limitations and future research

This study has several limitations that pave the way for future scholarly inquiry. First, although structural equation modeling provides strong inferential support for the hypothesized pathways, the cross-sectional nature of the survey data precludes absolute causal claims, necessitating longitudinal studies to capture how students' interaction paradigms evolve over time. Second, because the sample was exclusively drawn from Chinese higher education institutions, cultural variables and specific institutional policies regarding AI may influence behavior. Future studies should replicate this model across diverse global educational contexts to help validate the cross-cultural universality of the asymmetrical mediation observed here. Third, our use of a purposive sampling technique, which required participants to have prior experience with GenAI, was necessary to ensure response validity. However, this approach may limit the generalizability of our findings to the broader student population, including novices or non-users of AI tools. Future research could employ random sampling to capture a more comprehensive spectrum of student experiences.

## 6 Conclusion

As higher education increasingly integrates generative artificial intelligence into learning environments, understanding the cognitive consequences of different interaction patterns has become increasingly important. The findings of this study suggest that the educational value of AI does not solely depend on the intensity of human-machine interaction, but rather on how learners regulate their cognitive engagement during AI-supported learning processes. While autonomous and efficiency-oriented AI use demonstrated a modest but significant contribution to knowledge application ability, deeper forms of human-machine synergy did not produce the expected cognitive benefits in this study. These findings indicate that extensive AI collaboration may not inherently promote deeper learning and may even introduce risks of excessive cognitive offloading when learners relinquish critical cognitive responsibilities to algorithmic systems. Ultimately, the findings reinforce the importance of preserving learners' cognitive agency in AI-assisted education and suggest that the future effectiveness of generative AI in higher education may depend more on pedagogical regulation than on technological sophistication alone. Therefore, higher education institutions should design structured curricular and assessment mechanisms that guide students to critically evaluate AI-generated outputs, maintain independent reasoning, and integrate AI use into reflective knowledge application activities.

## Data Availability

The original contributions presented in the study are included in the article/supplementary material, further inquiries can be directed to the corresponding author.
